# Paramagnetic rim lesions as a biomarker to discriminate between multiple sclerosis and cerebral small vessel disease

**DOI:** 10.3389/fneur.2024.1429698

**Published:** 2024-07-16

**Authors:** Zhibao Zhu, Yuanyuan Zhang, Chun Li, Wenliang Guo, Zhili Chen, Wei Chen, Shaowu Li, Ning Wang, Xiaochun Chen, Ying Fu

**Affiliations:** ^1^Department of Neurology, Fujian Medical University Union Hospital, Fuzhou, Fujian, China; ^2^Department of Neurology, Fujian Institute of Neurology, The First Affiliated Hospital, Fujian Medical University, Fuzhou, Fujian, China; ^3^Fujian Key Laboratory of Molecular Neurology, Institute of Neuroscience, Fujian Medical University, Fuzhou, Fujian, China; ^4^Department of Neurology, National Regional Medical Center, Binhai Campus of the First Affiliated Hospital, Fujian Medical University, Fuzhou, China; ^5^Department of Neurology, China National Clinical Research Center for Neurological Diseases, Beijing Tiantan Hospital, Capital Medical University, Beijing, China

**Keywords:** paramagnetic rim lesions, multiple sclerosis, cerebral small vessel disease, magnetic resonance imaging, biomarker

## Abstract

**Background:**

Multiple sclerosis (MS) and Cerebral Small Vessel Disease (CSVD) exhibit some similarities in Magnetic resonance imaging (MRI), potentially leading to misdiagnosis and delaying effective treatment windows. It is unclear whether CSVD can be detected with Paramagnetic Rim Lesions (PRL), which is special in MS.

**Objective:**

We aimed to investigate whether PRL can serve as a neuroimaging marker for discriminating between MS and CSVD.

**Methods:**

In this retrospective study, 49 MS and 104 CSVD patients underwent 3.0 T Magnetic resonance imaging (MRI). Visual assessment of 37 MS patients and 89 CSVD patients with or without lacunes, cerebral microbleeds (CMBs), enlarged perivascular spaces (EPVS), white matter hyperintensity (WMH), central vein sign (CVS), and PRL. The distribution and number of PRL were then counted.

**Results:**

Our study found that PRL was detected in over half of the MS patients but was entirely absent in CSVD patients (78.38 vs. 0%, *p* < 0.0001), and PRL showed high specificity with good sensitivity in discriminating between MS and CSVD (sensitivity: 78.38%, specificity: 100%, AUC: 0.96).

**Conclusion:**

Paramagnetic Rim Lesions is a special imaging feature in MS, absent in CSVD. Detection of PRL can be very helpful in the clinical management of MS and CSVD.

## Introduction

Multiple sclerosis (MS) is a debilitating neuroimmune disorder that triggers the body’s immune system to attack the central nervous system (CNS), slowly robbing patients of their physical mobility (1, 2). Cerebral small vessel disease (CSVD) refers to a group of pathological processes with various causes that affect capillaries, small arteries and small veins in the brain (3).

It is important to remember that, despite having different underlying mechanisms (2, 4), some clinical cases may be misdiagnosed as MS and CSVD (5, 6), making accurate diagnosis and timely treatment difficult. As we know, brain MRI scans can reveal several features closely associated with CSVD (3, 7), such as lacunes, cerebral microbleeds (CMBs), enlarged perivascular spaces (EPVS), and white matter hyperintensity (WMH). Recent studies have reported that several magnetic resonance imaging (MRI) imaging features in MS patients show similarities to those in CSVD patients, including the presence of lacunes (8, 9), CMBs (10), and EPVS (11, 12). To prevent misdiagnosis and unwarranted treatment, the identification of novel neuroimaging markers to differentiate between MS and CSVD is essential.

Compared with CSVD (13), WMH is also a common MRI feature of MS (14). Following the acute phase of inflammatory demyelination during the onset of MS lesions, some chronic lesions are identified as Paramagnetic Rim Lesions (PRL) (15). Pieces of research from MRI and histopathology has revealed that these chronic lesions are marked by a characteristic paramagnetic rim (16, 17), a specific imaging feature that can be employed for MS diagnosis (18).

However, the feasibility of detecting PRL within CSVD-related WMH remains uncertain. There has been no comprehensive comparative assessment of PRL between MS and CSVD.

Hence, this study aimed to explore the potential utility of PRL as a neuroimaging marker for discriminating between MS and CSVD.

## Materials and methods

### Study design and subjects

The study protocol and informed consent procedures received approval from the regional ethics review boards of TianTan Hospital of Capital Medical University. All the participants provided written informed consent to participate in this study. This retrospective study analyses data from patients diagnosed with MS or CSVD collected between 2016 and 2017.

Diagnoses were made by two neuroradiologists (YF and SL, each with 10 years of experience in MRI processing). MS patients were diagnosed based on the 2017 revisions of the McDonald criteria (19), while CSVD patients were diagnosed based on clinical symptoms combined with typical MRI imaging features (7). Patients falling outside of these categories, those presenting MRI findings of CSVD along with other brain diseases (e.g., acute cerebral infarction), incomplete MRI sequences and data, or those under 18 years old, were explicitly excluded.

### MRI acquisition

All imaging acquisitions were performed on the same Siemens 3.0 T Magnetom Prisma Fit MRI scanner (Siemens Healthcare, Erlangen, Germany) equipped with a 64-channel head coil. The imaging protocol comprised: (1) A 3D T1-weighted sequence (TR = 2,300 ms, TE = 2.32 ms, TI = 900 ms, flip angle = 8°, FOV = 240 × 240 mm^2^, voxel size = 0.9 × 0.9 × 0.9 mm^3^, slice thickness = 0.9 mm, number of slices = 256); (2) A T2-weighted sequence (TR = 5,000 ms, TE = 105 ms, flip angle = 150°, FOV = 199 × 220 mm^2^, voxel size = 0.5 × 0.5 × 4 mm^3^, slice thickness = 3 mm, number of slices = 33); (3) A T2-weighted fluid-attenuated inversion recovery (FLAIR) sequence (TR = 9,000 ms, TE = 81 ms, TI = 2,500 ms, flip angle = 150°, FOV = 220 × 220 mm^2^, voxel size = 0.7 × 0.7 × 6.5 mm^3^, slice thickness = 5 mm, number of slices = 20); (4) A susceptibility-weighted imaging (SWI) sequence (TR = 29 ms, TE = 20 ms, flip angle = 15°, FOV = 192 × 220 mm^2^, voxel size = 0.5 × 0.5 × 1.2 mm^3^, slice thickness = 1.5 mm, number of slices = 37).

### MRI analysis

#### Imaging features of CSVD and MS

Two experienced neuroradiologists (ZZ and WG, each with 3 years of experience in MRI processing), who were blinded to the patient’s clinical information, visually assessed imaging features according to the Standards for Reporting Vascular Changes on Neuroimaging (STRIVE) guidelines (13) for CSVD and MS patients, and established MRI guidelines (14, 20–22) for MS patients. The scoring for total CSVD, ranging from 0 to 6, was determined based on individual imaging features (23): 1 point for the presence of (a) any lacunes; (b) 1–4 CMBs; (c) moderate to severe BG-EPVS (> 20); (d) moderate WMH (range 3–4) (total periventricular + deep WMH Fazekas score). Additionally, two points were assigned for the presence of (a) ≥ 5 CMBs; (b) severe WMH (range 5–6) (total periventricular + deep WMH Fazekas score). In cases of inconsistent evaluations by the two neuroradiologists, a third neuroradiologist (YF) decided.

#### Identification of CVS

The central vein sign (CVS) was assessed on SWI images for all nonconfluent WMH extending for at least 3 mm in the shortest diameter, following North American Imaging in MS Cooperative (NAIMS) criteria (24). Three neuroradiologists (CL, ZC, and WC, each with 3 years of experience in MRI processing) evaluated the presence of CVS in MS and CSVD without clinical data and tested the Interrater consistency. Three observers ensured consensus in cases of inconsistency in PRL identification through collective discussion. We added this partial data to the Supplementary material.

#### Identification of PRL

Following MRI acquisition, a proprietary reconstruction algorithm automatically processed susceptibility-weighted images to generate axial filtered-phase SWI images. Subsequently, all images were converted to NIfTI format using the dcm2niix tool (Chris Rorden, Neuroimaging Tools & Resources Collaboratory). Registration of the images (T1, T2, T2 FLAIR, and SWI) with the phase image was achieved utilizing the core registration algorithm in SPM12.^1^

According to medical terminology, PRL is characterized by displaying a hypointense rim on phase image, has internal isointense or slight hyperintense to extralesional white matter, and visibility on at least three consecutive sections (25, 26). Due to our phase image is from a left-handed system, wherein gray matter appears hyperintense, based on the Handedness of MRI Systems Imaging Tip (27). As a result, the PRL on our phase image displays a hyperintense rim and has internal isointense or slight hypointense to extralesional white matter. To assess the truth of PRL (Supplementary Figure S2), we used a visual assessment by combining the T1, T2, T2 FLAIR, and SWI images with phase image. The distribution and number of PRL were then counted. It was evaluated by five neuroradiologists (CL, ZLC, WLG, YZ, and WC, each with 3 years of experience in MRI processing) without clinical data and tested for consistency. Five observers ensured consensus in cases of inconsistency in PRL identification through collective discussion. Lesions less than 3 mm in diameter were excluded due to being too small.

### Statistical analysis

We conducted statistical analyses using SPSS (version 26.0, IBM) and Prism (version 7.0, GraphPad). Our results are presented as means (±SD) for normally distributed data and medians (IQR) for data with non-normal distribution. As appropriate, demographic and MRI differences were evaluated using the Mann–Whitney U test or the Student’s *t*-test, along with Fisher’s exact or Chi-square test. Interrater reliability for assessing lacunes, CMBs, BG-EPVS, WMH, CVS, and PRL was determined using Cohen’s κ and Kendall’s w statistics. The receiver-operating characteristic (ROC) curve and PRL distributions were analyzed and visualized using the Python (version 3) language modules; Sklearn, Matplotlib, and Seaborn. *p* values <0.05 were considered statistically significant.

## Results

### Clinical patients characteristics

**Figure 1:**
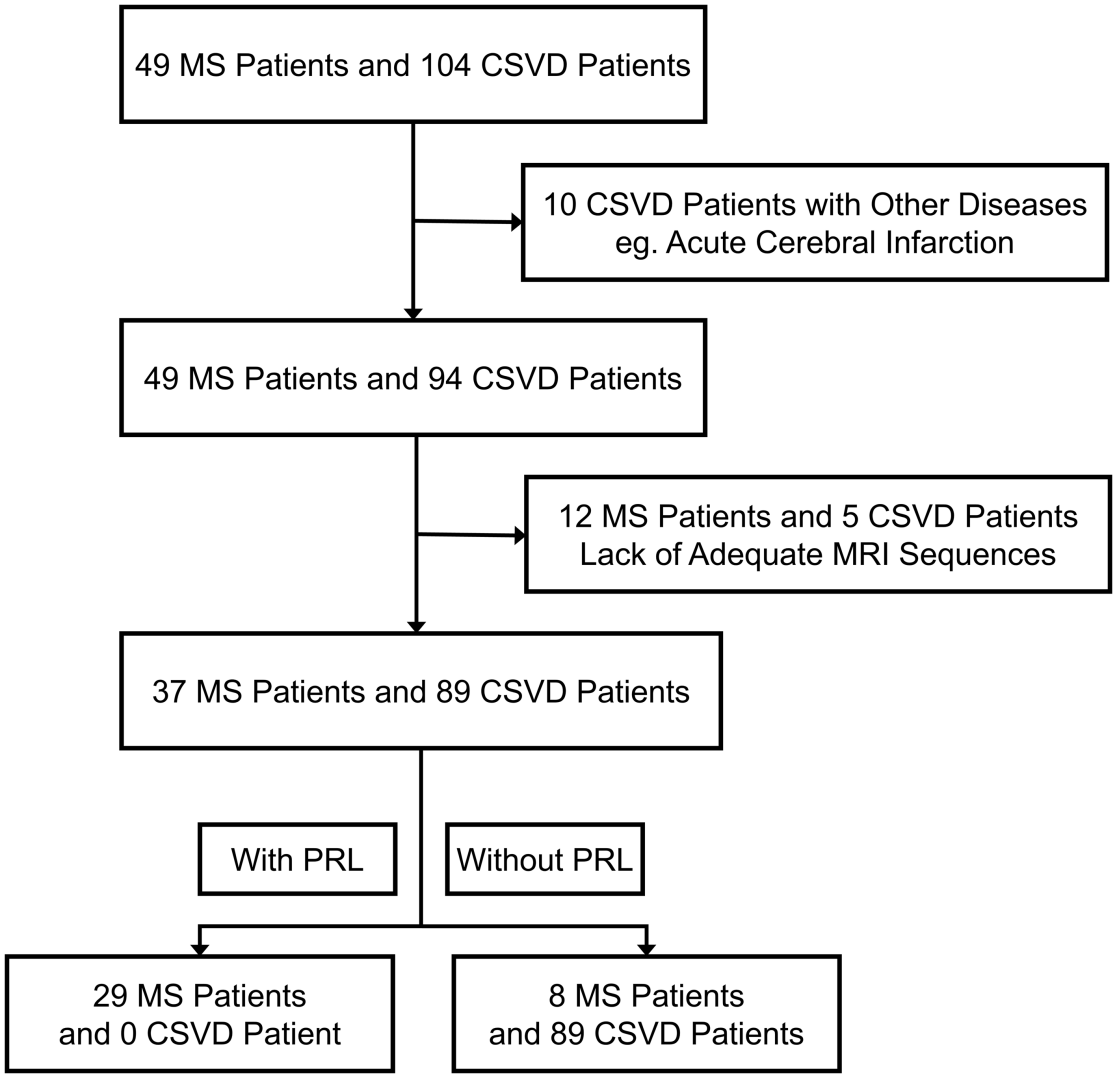
Trial profile. MS, Multiple sclerosis; CSVD, Cerebral small vessel disease; PRL, Paramagnetic rim lesions.

Our study assessed the eligibility of 153 MS and CSVD cases for inclusion (Figure 1). After applying specific criteria, we retained 126 patients for analysis. Exclusion primarily resulted from inadequate quality MRI data, affecting 17 participants. Ten participants were excluded due to other brain diseases evident on MRI. Demographic and characteristic details of study participants, comprising 37 MS and 89 CSVD patients, are presented. Due to the pathogenetic characteristics of MS, our patients with MS had a younger age of onset (35.00 ± 10.51 vs. 54.54 ± 13.14, *p* < 0.0001) and were predominantly female (75.68 vs. 51.69%, *p* = 0.0127) compared to patients with CSVD (Table 1).

**Table 1:** Demographic and radiologic characteristics of the study population.

Characteristics	MS (*n* = 37)	CSVD (*n* = 89)	*p* value
Age, year, mean (SD)	35.00 (10.51)	54.54 (13.14)	^<0.0001
Female, *n* (%)	28 (75.68)	46 (51.69)	^#^0.0127
Presence of lacunes, *n* (%)	3 (8.11)	52 (58.43)	^*^<0.0001
BG-EPVS (*N* > 20), *n* (%)	2 (5.41)	40 (44.94)	^*^<0.0001
Presence of WMH, *n* (%)	30 (81.08)	72 (80.90)	^#^0.9811
WMH score, 1 point, *n* (%)	11 (29.73)	25 (28.09)	^#^0.8528
WMH score, 2 points, *n* (%)	19 (51.35)	47 (52.81)	^#^0.8814
Presence of CMBs, *n* (%)	4 (10.81)	49 (55.06)	^*^<0.0001
1 (1–4 CMBs), *n* (%)	4 (10.81)	22 (24.72)	^*^0.0939
2 (≥ 5 CMBs), *n* (%)	0 (0)	27 (30.34)	^*^<0.0001
Total CSVD score, median (IQR)	2 (1–2)	3 (2–5)	^$^<0.0001
Presence of PRL, *n* (%)	29 (78.38)	0 (0)	^*^<0.0001

^^^Student’s *t*-test. ^#^Chi-square test. ^*^Fisher’s exact test. ^$^Mann–Whitney U test. MS, Multiple sclerosis; CSVD, Cerebral small vessel disease; BG-EPVS, Enlarged perivascular spaces (EPVS) in the basal ganglia; CMBs, Cerebral microbleeds; WMH, White matter hyperintensity; IQR, Interquartile range; PRL, Paramagnetic rim lesions.

### Inter-observer agreement

The coefficient of concordance for lacunes, CMBs, BG-EPVS, and WMH was substantial and almost perfect, ranging between 0.65 and 0.84. For CVS identification, two observers exhibited substantial agreement with κ = 0.769 for MS and κ = 0.786 for CSVD. Concordance of CVS identification by three observers was assessed using Kendall’s w statistic, yielding the following results: Kendall’s *w* = 0.714 for MS and CSVD (Supplementary Table S2). For PRL identification, two observers exhibited substantial agreement with κ = 0.77 for MS and κ = 0.66 for CSVD. Concordance of PRL identification by three to five observers was assessed using Kendall’s w statistic, yielding the following results: Kendall’s *w* = 0.90 for MS and 0.83 for CSVD by three observers, Kendall’s *w* = 0.83 for MS and 0.75 for CSVD by four observers, and Kendall’s *w* = 0.80 for MS and 0.60 for CSVD by five observers (Table 2).

**Table 2:** Interrater agreement for lesions on MRI.

	Cohen’s κ					Kendall’s *w*		
	Lacunes (*n* = 2)	BG-EPVS (*n* = 2)	WMH (*n* = 2)	CMBs (*n* = 2)	PRL (*n* = 2)	PRL (*n* = 3)	PRL (*n* = 4)	PRL (*n* = 5)
MS	0.79	0.65	0.78	0.84	0.77	0.90	0.83	0.80
CSVD	0.75	0.73	0.76	0.77	0.66	0.83	0.75	0.60

*n*, Number of neuroradiologists; MS, Multiple sclerosis; CSVD, Cerebral small vessel disease; BG-EPVS, Enlarged perivascular spaces (EPVS) in the basal ganglia; WMH, White matter hyperintensity; CMBs, Cerebral microbleeds; and PRL, Paramagnetic rim lesions.

### Comparison of MRI imaging features between MS and CSVD

The results indicate that CSVD patients encompassed a more significant occurrence of lacunes (58.43%, *p* < 0.0001), BG-EPVS (44.94%, *p* < 0.0001), CMBs (55.06%, *p* < 0.0001), and higher total CSVD scores (3, [IQR] 2–5 vs. 2, [IQR] 1–2, *p* < 0.0001) than MS patients (Table 1). There was no significant difference in WMH between CSVD and MS patients (80.90 vs. 81.08%, *p* = 0.9811), consistent with the fact that WMH was common in both (Table 1).

**Figure 2:**
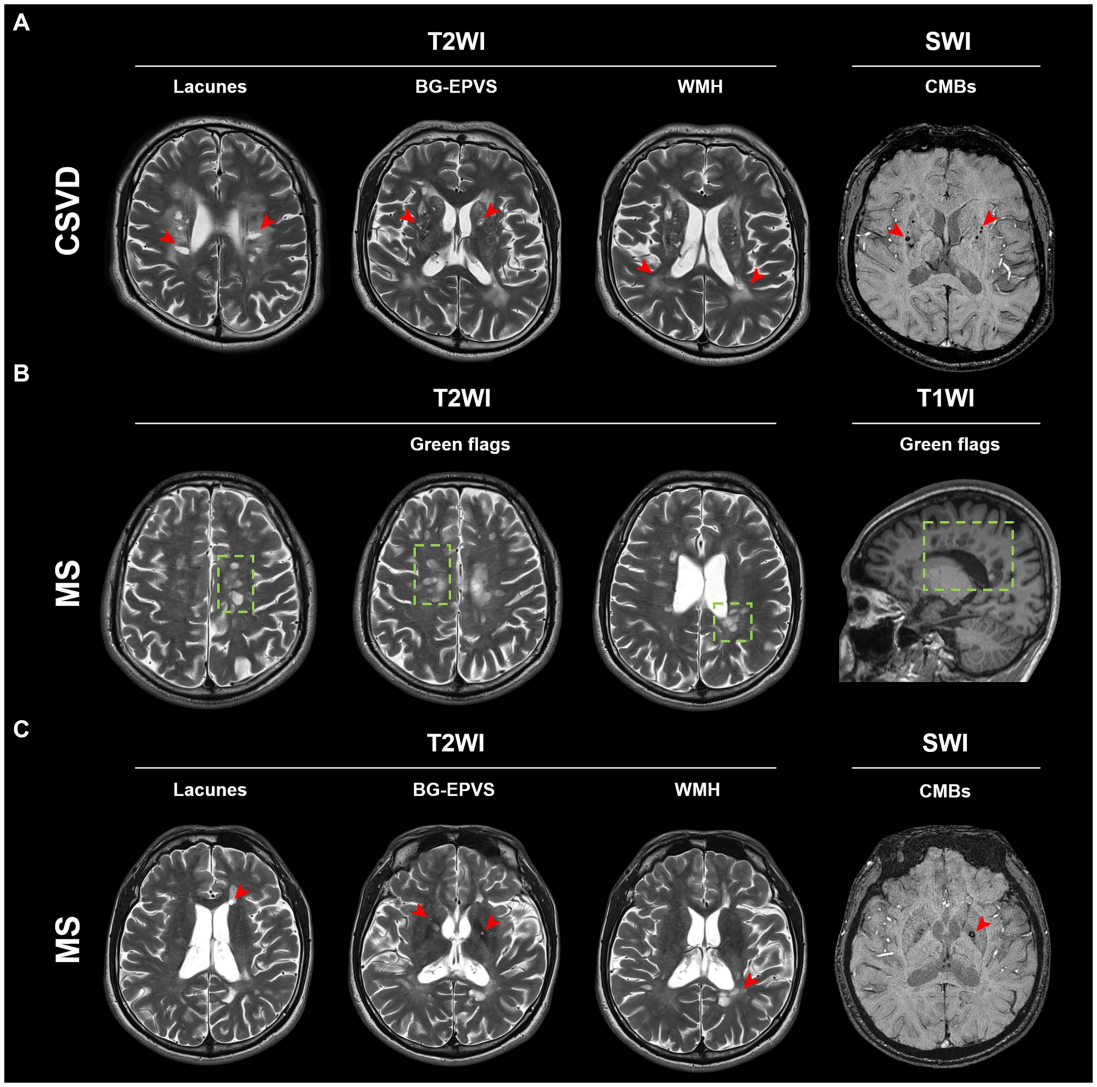
Typical MRI imaging features of MS and CSVD. **(A)** Typical lesions of lacunes, BG-EPVS, WMH, and CMBs (red arrow) in a CSVD patient. **(B)** Typical green flags in an MS patient. Green flags: periventricular lesions suggestive of multiple sclerosis; or periventricular lesions perpendicular to the corpus callosum (“Dawson’s fingers”). **(C)** Lacunes, BG-EPVS, WMH, and CMBs (red arrow) can also be found in this MS patient. BG-EPVS, Enlarged perivascular spaces (EPVS) in the basal ganglia; WMH, White matter hyperintensity; CMBs, Cerebral microbleeds; MS, Multiple sclerosis; and CSVD, Cerebral small vessel disease.

Although the aforementioned MRI imaging features were prevalent in CSVD patients (Figure 2A), some MS patients also showed similar imaging features (Figures 2B,C). This makes it challenging to discriminate between MS and CSVD on MRI. We found that PRL was present in more than half of the cases in the subgroup of MS patients with CSVD imaging features (Table 3). However, there were no significant differences in CSVD imaging features between MS patients with and without PRL (Supplementary Table S1).

**Table 3:** The presence of PRL in MS patients with CSVD imaging features.

	Lacunes (*N* ≥ 1)	BG-EPVS (*N* > 20)	WMH (≥ 1 point)	CMBs (*N* ≥ 1)
Cases with at least one PRL, ratio (%)	2/3 (66.67)	2/2 (100.00)	26/30 (86.67)	2/4 (50.00)

*n*, Number of lesions; MS, Multiple sclerosis; CSVD, Cerebral small vessel disease; PRL, Paramagnetic rim lesions; BG-EPVS, Enlarged perivascular spaces (EPVS) in the basal ganglia; WMH, White matter hyperintensity; and CMBs, Cerebral microbleeds.

### Identification and diagnostic efficacy of CVS between MS and CSVD

In our study, we found that CVS was detected in both MS and CSVD (Supplementary Figure S1A). The percentage of cases with CVS is higher in MS than in CSVD (86.49 vs. 26.97%, *p* < 0.0001) (Supplementary Figure S1B; Supplementary Table S3). Furthermore, the ROC result demonstrated high specificity but rather low sensitivity of CVS in discriminating between MS and CSVD (sensitivity: 57.14%, specificity: 92.86%, AUC: 0.75) (Supplementary Figure S1C).

### Identification and distribution of PRL

**Figure 3:**
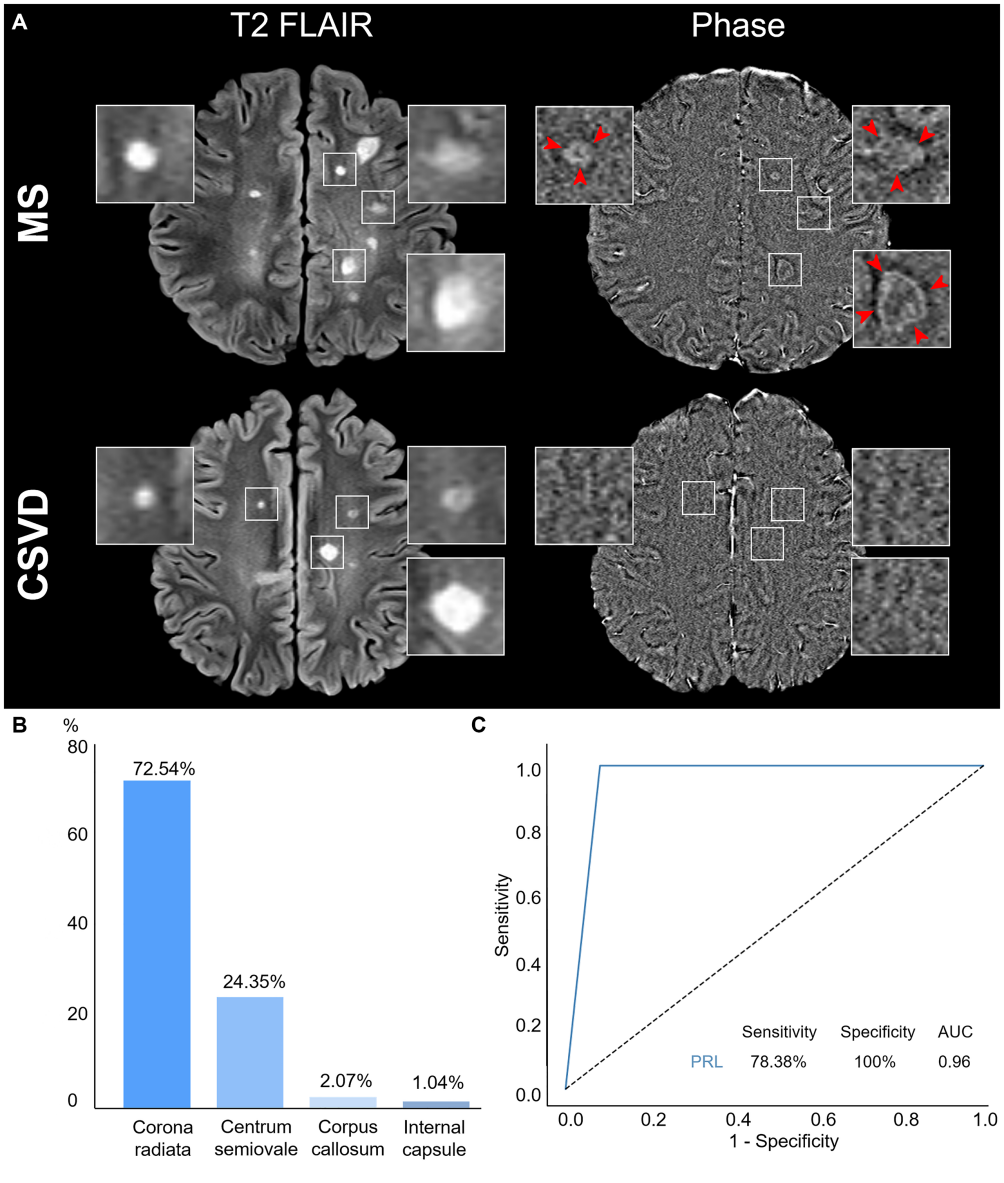
Characteristics and diagnostic efficacy of PRL. **(A)** Representative PRL displays a hyperintense rim and has internal isointense or slight hypointense to extralesional white matter. WMH was shown in both MS and CSVD Axial T2 FLAIR, while PRL was detected only in of MS phase image (red arrow) but not in CSVD. **(B)** Distributions of PRL in MS patients. **(C)** The ROC curve of PRL discrimination between MS and CSVD. PRL, Paramagnetic rim lesions; WMH, White matter hyperintensity; MS, Multiple sclerosis; CSVD, Cerebral small vessel disease; ROC, Receiver operating characteristic; and AUC, Area under the curve.

We utilized a combination of multiple image sequences for visual assessment, which revealed that PRL was exclusively observed in MS within the corresponding WMH areas on phase image (Figure 3A). Remarkably, our study detected PRL in over half of the MS cases but not in CSVD (78.38 vs. 0%, *p* < 0.0001) (Table 1).

Moreover, among MS patients with PRL, the distribution pattern indicated a higher prevalence in the corona radiata, followed by the centrum semiovale, and less frequently in the corpus callosum and the internal capsule (Figure 3B).

### Diagnostic efficacy of PRL between MS and CSVD

To assess the utility of PRL as an imaging marker for differentiating between MS and CSVD, we calculated the area under the curve (AUC) using ROC analysis. Our results demonstrated high specificity with good sensitivity of PRL in discriminating between MS and CSVD (sensitivity: 78.38%, specificity: 100%, AUC: 0.96) (Figure 3C).

## Discussion

In this study, we evaluated the diagnostic utility of PRL observed on 3.0 T MRI in discriminating between MS and CSVD patients. We observed a high percentage of PRL in lesions of MS patients but its absence in CSVD patients. Furthermore, PRL showed high specificity with good sensitivity in discriminating between MS and CSVD. These promising results were obtained using routine clinical imaging data.

Current diagnostic criteria (19) and guidelines (20) for MS acknowledge the challenge of reliably differentiating WMH using conventional MRI (28). Our results corroborate this, as both MS and CSVD patients exhibited WMH on MRI, accounting for over 80% of cases. Notably, previous research has identified other CSVD imaging features, including lacunes (8, 9), CMBs (10), and EPVS (11, 12), in some MS cases, which can contribute to misdiagnosis and treatment delays. Some studies have used CVS to explore the difference between MS and non-MS (24, 29). However, it is important to note that CVS is not specific to MS and can be challenging to observe in clinical 3.0 T MRI compared with ultra-high-field at 7.0 T (30). Our results on the CVS assessment between MS and CSVD suggest that CVS is not unique to MS (Supplementary material S1).

Compared to CVS, PRL is a specific imaging feature in MS, suggesting its potential to be a biomarker for discriminating between MS and CSVD. Earlier studies explored PRL identification in both MS and vascular diseases. One of which included 32 patients with MS and vascular diseases but only one case of CSVD, using FLAIR* at 7.0 T, failed to detect PRL in vascular lesions (31). Later, an international multicenter 3.0 T MRI study on PRL included 22 individuals suffering from non-inflammatory neurological disorders (NIND), which involved small vessel disease and migraine, using 3D T2-FLAIR image and submillimeter isotropic 3D segmented T2*-weighted EPI to provide phase image, and also found the presence of PRL was to be absent in those with NIND (26). The limited number of CSVD cases in these studies makes it difficult to draw clear conclusions. Compared with the above, we similarly find that PRL was only present in MS but not in CSVD, and PRL accounted for a high proportion of MS (78.38 vs. 0%, *p* < 0.0001). This is probably because our methodological enhancements, integrating more patients, employed filtered-phase SWI images with higher signal-to-noise ratios in combination with multiple image sequences for PRL identification and engaged five neuroradiologists in the assessment. Additionally, although the observed PRL was similar to previous 3.0 T MRI studies (26, 32, 33), our study featured five independent observers with repeated assessments in cases of disagreement, further reinforcing the accuracy of visual assessments.

According to the consensus (21, 22), we can identify PRL in most cases, but misdiagnosis can occur in some conditions. To address this issue, our study lists several illustrative examples of determining whether PRL is true or false, which can be invaluable in clinical practice (Supplementary Figure S2). If the MRI is a left-handed system (27), a chronic active lesion can be observed as a hyperintense rim on phase image, the region corresponding to the lesion is a focal WMH with approximately coincident edges on T2 FLAIR, and PRL typically appears hypointense on T1 and hyperintense on T2, occasionally displaying a hypointense rim on SWI. When the region corresponding to the lesion in the different sequences does not have a lesion or the lesion signals are inconsistent with the above, we must exclude it even if it appears as a typical PRL feature on phase image.

The insignificant association between CSVD imaging features and the development of PRL in our MS patients is attributed to the fact that PRL arises from neuroinflammatory pathologies, including inflammatory demyelination (17, 34), and microglia inflamed in MS (MIMS) (35). Most MS lesions are centered on small parenchymal veins (36), while the CSVD imaging features in MS imply additional vascular impairment (37, 38). We did not find PRL in CSVD, suggesting that WMH in CSVD (4) may be linked to vascular injury (39, 40), which has been referred to as WMH of presumed vascular origin (7).

Consistent with previous reports (31, 41), our PRL distributions predominantly occurred in the regions of corona radiata and centrum semiovale, emphasizing the association of PRL with regions of brain inflammation from an imaging perspective. This finding further supports microglia activation as a significant contributor to the pathogenesis of periventricular white matter damage (PWMD) (42, 43). Therefore, the PRL search should focus on these brain regions.

In MS, chronic lesion activity can also be detected by slowly expanding lesions (SEL) (44, 45), and The detection rate of SEL is much higher than PRL (45). If PRL is not detected on MRI, it may be a novel strategy to differentiate MS from CSVD based on SEL identification. SEL is mainly distributed around the ventricle and centrum semiovale (44), but the WMH of CSVD will also appear in the above areas (13). Considering the lack of specificity in SEL identification, exploring the feasibility of using SEL to discriminate between MS and CSVD is necessary.

## Limitations

Nevertheless, it is a retrospective study, and detailed information is incomplete. Our study has other certain limitations, including a relatively small sample size, possible selection bias, and the use of MRI from a single manufacturer. Different scanners, field strengths, equipment upgrades, and changes in acquisition parameters may affect PRL assessment (32). Additionally, CSVD patients without PRL lacked pathologic validation support.

## Conclusion

Paramagnetic Rim Lesions is a special imaging feature of MS but is not present in CSVD. When encountering patients with suspected MS displaying CSVD imaging features that cannot be reliably identified using conventional imaging criteria, the detection of PRL can confirm an MS diagnosis, facilitating timely intervention with medication. This discovery provides promising imaging evidence for MS and CSVD clinical management.

## Data availability statement

The raw data supporting the conclusions of this article will be made available by the authors, without undue reservation.

## Ethics statement

The studies involving humans were approved by TianTan Hospital of Capital Medical University. The studies were conducted in accordance with the local legislation and institutional requirements. The participants provided their written informed consent to participate in this study.

## Author contributions

ZZ: Conceptualization, Data curation, Formal analysis, Investigation, Methodology, Project administration, Software, Supervision, Validation, Visualization, Writing – original draft, Writing – review & editing. YZ: Conceptualization, Data curation, Formal analysis, Investigation, Methodology, Project administration, Software, Supervision, Validation, Visualization, Writing – original draft, Writing – review & editing. CL: Conceptualization, Data curation, Investigation, Methodology, Project administration, Supervision, Validation, Visualization, Writing – original draft, Writing – review & editing, Formal analysis. WG: Data curation, Methodology, Validation, Visualization, Writing – original draft. ZC: Data curation, Methodology, Formal analysis, Software, Writing – review & editing. WC: Methodology, Writing – review & editing, Investigation, Project administration. SL: Methodology, Project administration, Writing – review & editing, Data curation, Formal analysis, Resources, Software. NW: Project administration, Resources, Writing – review & editing, Conceptualization, Supervision, Writing – original draft. XC: Conceptualization, Resources, Writing – original draft, Data curation, Formal analysis, Funding acquisition, Writing – review & editing. YF: Conceptualization, Data curation, Formal analysis, Writing – review & editing, Funding acquisition, Investigation, Methodology, Project administration, Resources, Software, Supervision, Validation, Visualization, Writing – original draft.

## Footnotes

^1^ http://www.fil.ion.ucl.ac.uk/spm/software/spm12
